# Proportion of Protrusion of Proximal Femoral Nail Antirotation 2 Over the Greater Trochanter in Pertrochanteric Femur Fracture: A Cross-Sectional Study

**DOI:** 10.7759/cureus.88777

**Published:** 2025-07-25

**Authors:** Sushil Mankar, Darshan Sharma, Nitin Pothare, Vismay V Harkare, Rahul H Sakhare, Suraj Maddewad, Parimal S Vairagade, Ronit P Dalvi, Vijay D Surve, Deep Patel Patel

**Affiliations:** 1 Orthopaedics and Traumatology, N.K.P. Salve Institute of Medical Sciences & Research Centre and Lata Mangeshkar Hospital, Nagpur, IND; 2 Spine Surgery, N.K.P. Salve Institute of Medical Sciences & Research Centre and Lata Mangeshkar Hospital, Nagpur, IND; 3 Orthopaedics, N.K.P. Salve Institute of Medical Sciences & Research Centre and Lata Mangeshkar Hospital, Nagpur, IND; 4 Orthopaedic Surgery, N.K.P. Salve Institute of Medical Sciences & Research Centre and Lata Mangeshkar Hospital, Nagpur, IND

**Keywords:** greater trochanter, hip fracture, intertrochanteric fracture, proximal femoral nail, proximal femur fracture

## Abstract

Introduction

Pertrochanteric femur fractures comprise approximately half of all hip fractures. The proximal femoral nail anti-rotation 2 (PFNA2), designed by Arbeitsgemeinschaft fur Osteosynthesefragen (AO), is an intramedullary device with a helical blade rather than a screw for better purchase in the femoral head. This modification in the proximal femur nail (PFN) was done to adapt to the Asian population and reduce nail-related complications in the Asian population.

Materials and methods

The study was conducted in a tertiary health center from November 2022 to November 2024 and included 40 subjects with pertrochanteric fractures operated on with PFNA2. Patients were followed up at six weeks and six months. Parker's ratio, nail protrusion height, and Harris Hip Score (HHS) were calculated.

Observations and results

The majority of the patients in the study belonged to the age group of 60-75 years (37.5%). Nail protrusion height shows wide variability, ranging from 0.00 mm to 29.30 mm, with a mean of 6.01 mm and a considerable standard deviation (SD) of 10.39 mm. Parker's ratio, postoperatively, had a mean of 0.41 with SD of 0.88, ranging from 0.18 to 0.63. HHS was used to perform a clinical evaluation for these patients. The associated p-value for the correlation between Parker's ratio and HHS was 0.002 at six weeks, which is less than the significance level of 0.05, indicating that the correlation is statistically significant. The correlation between nail protrusion height and HHS was calculated by Pearson's correlation coefficient, which at six weeks was 𝑟=−0.210, indicating there was a negative correlation between nail protrusion height and HHS, so as the nail protrusion height increases, the HHS tends to decrease.

Conclusion

Pertrochanteric femur fracture is common in day-to-day life, with a bimodal age distribution. Our study concludes that PFNA2 is an excellent implant for the treatment of the pertrochanteric femur fracture. All the patients had an excellent follow-up score. A few patients, mainly female patients, had significant nail protrusion over the greater trochanter, with a higher Parker’s ratio, and had mild lateral hip pain. The pain was not significant and did not affect the patient’s day-to-day activities. This could be improved with the central placement of the helical blade. We do not recommend any changes in nail design; rather, we suggest proper helical blade placement to avoid nail protrusion. These variations due to sex and age should be studied further to establish any changes in implant design. Further studies with a bigger sample size and longer follow-up should be carried out for more definite results.

## Introduction

A pertrochanteric fracture starts anywhere on the greater trochanter laterally and exits the medial cortex either proximal or distal to the lesser trochanter [1]. They are a type of extracapsular fracture that can involve both the greater and lesser trochanter [2]. These fractures account for approximately half of all hip fractures due to low-energy mechanisms [3].

Pertrochanteric fractures are more commonly seen in women of higher age groups than in men. According to the Singh Index, only 49% women and 63% men have a good bone structure at the time of trauma [4]. These fractures occur in characteristic populations with risk factors including older age, female sex, osteoporosis, a history of trauma, and gait abnormality [5].

The proximal femoral nail antirotation 2 (PFNA2), designed by Arbeitsgemeinschaft fur Osteosynthesefragen (AO), is an intramedullary device with a helical blade rather than a screw for better purchase in the femoral head, as was tested in a clinical study [6]. The PFNA2 design has three modifications to the PFNA for Asian anatomic characteristics: (i) The proximal nail diameter was decreased from 17 mm to 16.5 mm, (ii) the mediolateral angle was lowered from 6° to 5°, and (iii) a flat proximal lateral surface was modified to avoid impingement of the femoral lateral cortex [6]. Protrusion of PFNA2 may affect clinical outcomes and patient satisfaction. Understanding the prevalence of protrusion and its impact on functional outcomes will guide clinicians in optimizing surgical approaches and postoperative care.

Conducting a study on the protrusion of PFNA2 over the greater trochanter in pertrochanteric femur fractures in the Indian population is justified because of the lack of specific data in this demographic group, the evolving landscape of fracture management, and the need for tailored approaches to enhance patient outcomes.

## Materials and methods

This was a cross-sectional study conducted at the Lata Mangeshkar Hospital, Nagpur, India, from November 2022 to November 2024. The study was approved by the Ethics Committee of N.K.P. Salve Institute of Medical Sciences & Research Centre and Lata Mangeshkar Hospital (approval number: 56/2022).

Participants

A total of 40 subjects with pertrochanteric fractures operated on with PFNA2 during the study period were included. Exclusion criteria were: (i) Patients with per trochanteric fractures with extension into the shaft of the femur, (ii) Polytrauma patients with ipsilateral lower limb fractures, (iii) Patients medically unfit for surgery, and (iv) Patients not willing to undergo surgery/participation in the study. Patients were followed up at six weeks and six months. Informed consent was given by all participants.

Preoperative workup

A detailed history was taken from the subjects, focusing on injury mechanisms, other associated injuries, comorbidities, and status before the injury occurred. A thorough evaluation of the patient's systemic or generalized conditions was done after diagnosis. Fracture limb examination along with local skin and soft tissue condition. Hematological investigations were done. The general condition was improved, and the patient was prepared for surgery and a suitable temporary stabilization of the fracture using skin traction with appropriate weights was done. Anesthesia fitness for surgery was obtained. Standard radiographs, including both anteroposterior and lateral views of both hips and the pelvis, were taken, and fracture anatomy was identified. Routine preoperative preparations were done. Similar antibiotics were administered at the start of the surgery.

Operative technique

The patient was taken on a fracture table in the supine position after receiving proper anesthesia. The affected leg of the patient was placed in an adducted position with a 10-15^o^ internal rotation. The hip joint was visualized anteroposteriorly and laterally with a C-arm. An attempt at closed reduction was made and checked under image intensification. In case of a failed attempt at closed reduction, indirect attempts were made via an open method. A 3-5 cm incision was taken 2 cm proximally to the greater trochanter tip, then with the help of a curved awl, entry was taken at the tip of the greater trochanter. An entry reamer was used (14 mm) for the accommodation of the proximal part of the nail.

**Figure 1:**
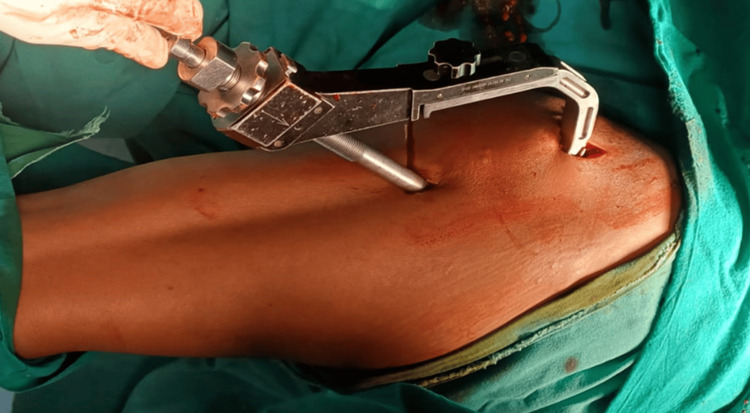
Insertion of a guide wire through the jig for the 8 mm helical blade.

**Figure 2:**
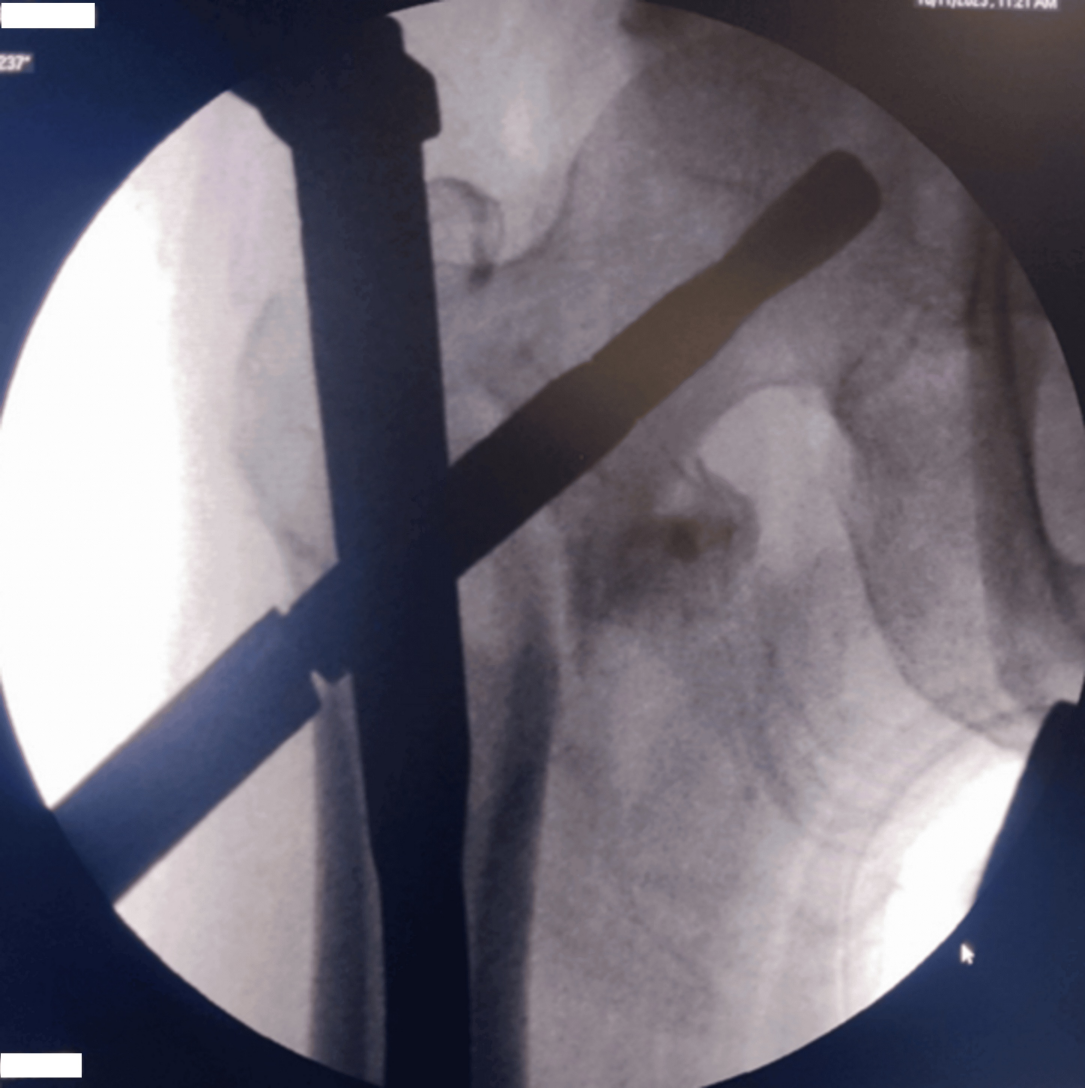
The C-arm picture of the insertion of the helical blade.

A nail of the pre-determined size was mounted on the appropriate jig and placed over the guide wire. The nail was then passed down. An aiming device that was attached to the jig was used to mark the entry for the helical blade. The guide wire was then removed (Figure 1). Reaming was done with a 6.4mm reamer mounted over the wire. An appropriately sized helical blade was inserted up to the subchondral level (Figure 2).

Using an appropriate guide, a stab incision was made on the lateral aspect of the thigh and was used for locking distally after drilling both cortices using a 4 mm drill bit and taking the measure of screw length using a depth gauge. Distal locking was done with a 4.9 mm bolt. Final images were taken, and closure was done in layers.

Postoperative management

Postoperatively, patients were advised non-weight-bearing mobilization for six weeks. Hip range of motion exercises, both active and passive, were instructed to the patient and done as tolerated by the patient. All patients were given a similar antibiotic regimen of intravenous antibiotics for three days postoperatively and then shifted to oral antibiotics till sutures were removed. Sterile dressing of the suture was done on day 2 and day 6 postoperatively.

Data collection

**Figure 3:**
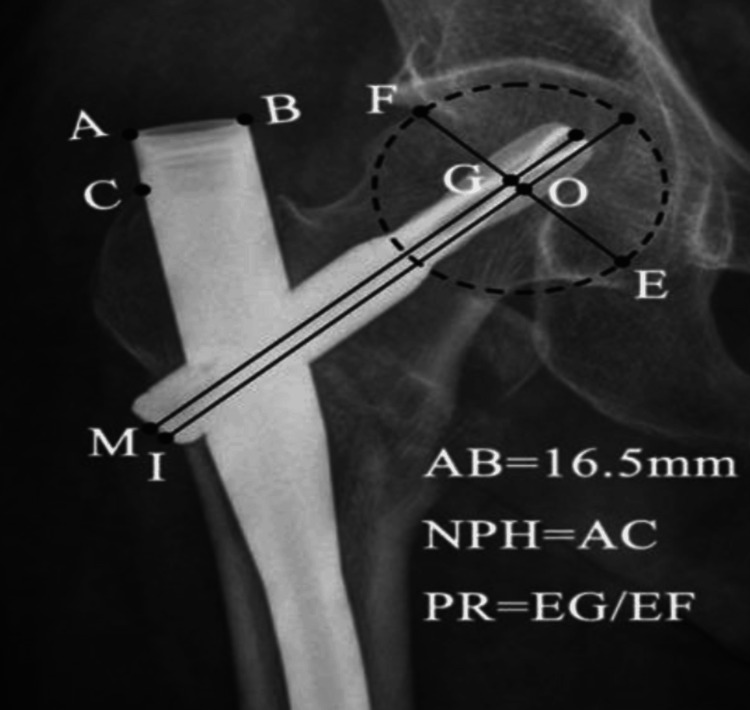
Parker’s ratio and nail protrusion height. A: proximal tip of nail on lateral side; B: proximal tip of nail on medial side; C: the lateral border of nail crossing the tip of the greater trochanter; O: the center of the femoral head line; I: head-neck axial passing the center of the femoral head; Line M: the helical blade axial line; Line EF: perpendicular to line I crossing point O; G: intersection point of lines EF and M; E, F: intersection points of line EF and the femoral head circle; NPH: nail protrusion height

The following parameters were included in data collection: (i) Parker’s ratio, which indicates the distance of the screw from the lower border of the femoral neck [6], (ii) Nail protrusion height (NPH), which is a measurement of the amount of protrusion of the femoral nail above the greater trochanter [6]. Measurements on the anteroposterior radiograph are shown in Figure 3. 

Follow-up

All the patients were followed up at six weeks and at six months. Radiographs and a clinical evaluation using the Harris Hip Score (HHS) were obtained during follow-up and recorded. The measurement values on the radiographs, as well as the values of the HHS, were measured by the same observer at all the follow-ups for all the patients.

Data and statistical analysis

Data analysis was done by measuring parameters by the Bone Ninja Application Version 5.0 (LifeBridge Health, Baltimore, Maryland). The HHS was used for the clinical evaluation. Age and sex-based evaluation was also done. Qualitative data were presented in the form of frequency and percentage. The association between qualitative variables was assessed using the Chi-square test with continuity correction for all 2 × 2 tables. Quantitative data were represented as mean ± SD. Continuous variables were correlated using Pearson's correlation coefficient. Appropriate statistical software (GNU PSPP version 2.0.1; https://www.gnu.org/software/pspp/) was used for statistical analysis. Graphical representations were prepared using MS Excel 365 (Microsoft Corporation, Redmond, Washington, United States).

## Results

A total of 40 subjects aged 45-90 years were included. Most patients were in the age group of 60-75 years (37.5%). Twenty-eight patients (70%) had stable fracture patterns, and 12 patients (30%) had unstable fracture patterns. Both male and female patients had a high incidence of stable fractures, with more female patients (85.7%) compared to male patients (61.5%). NPH shows wide variability, ranging from 0.00 mm to 29.30 mm, with a mean of 6.01 mm and a considerable SD of 10.39 mm.

Parker's ratio, postoperatively, has a mean of 0.41 with a SD of 0.88, ranging from 0.18 to 0.63. Clinical evaluation for these patients was done by HHS. Immediately postoperatively, the mean HHS was 32.69, with slight variability and ranging from 31.0 to 34.2. At six weeks postoperative, the mean score significantly increased to 80.32, with a moderate SD and a range of 78.0-84.0. By six months, the mean score further improved to 91.81, with a narrower SD and a range of 90.0 to 93.0, suggesting ongoing recovery and enhanced hip functionality over time (Table 1).

**Table 1:** Descriptive Statistics of Harris Hip Score at different intervals.

Harris Hip Score	Mean	Std. Deviation	Minimum	Maximum
Postoperative Score	32.69	0.93	31.0	34.2
6 weeks	80.32	1.44	78.0	84.0
6 months	91.81	0.78	90.0	93.0

**Figure 4:**
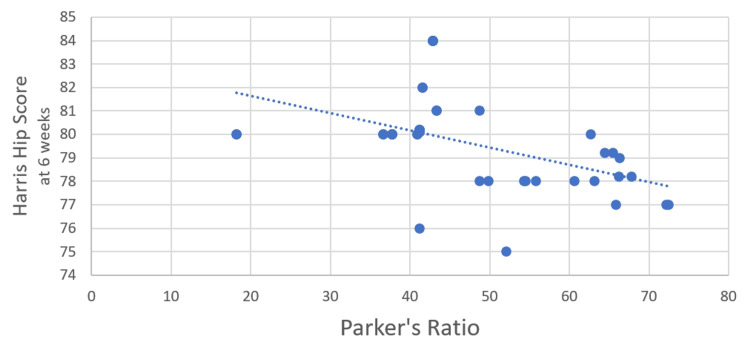
Correlation between Parker's raio and Harris Hip Score (6 weeks)

The correlation between Parker’s ratio and the HHS at six weeks post surgery was assessed using Pearson’s correlation coefficient. The analysis yielded a Pearson’s correlation coefficient 𝑟=−0.485, indicating a moderate negative correlation between these two variables. This suggests that higher values of Parker’s ratio are associated with lower HHS at six weeks post surgery, and vice versa. The p-value for this correlation was 0.002, providing strong evidence that the negative correlation between Parker’s ratio and the HHS at six weeks is statistically significant (Figure 4).

**Figure 5:**
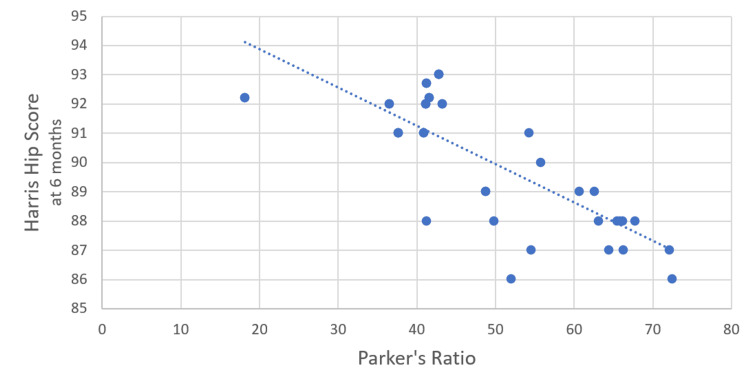
Correlation between Parker’s ratio and Harris Hip Score (6 months).

The correlation between Parker’s ratio and the HHS at six months postoperatively was calculated using Pearson’s correlation coefficient. The results showed a Pearson’s correlation coefficient 𝑟=−0.777, indicating a strong negative correlation between these two variables (Figure 5).

**Figure 6:**
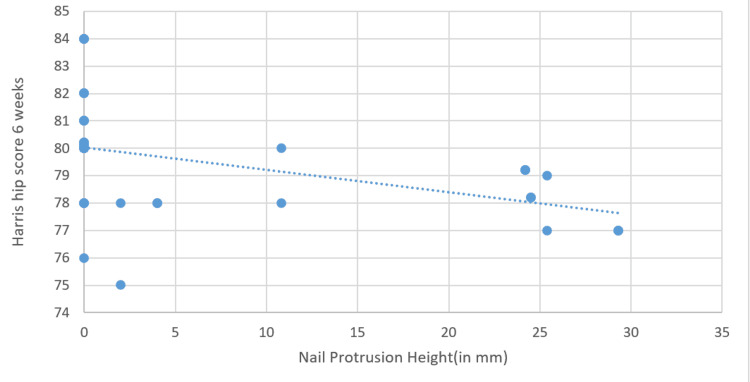
Correlation between nail protusion height and Harris Hip Score (6 weeks)

Pearson's correlation coefficient at six weeks was 𝑟=−0.210, indicating a weak negative correlation. This suggests a slight tendency for the Harris hip score to decrease as NPH increases, but the relationship is not strong. The P-value of 0.193 indicates that this correlation is not statistically significant (Figure 6).

**Figure 7:**
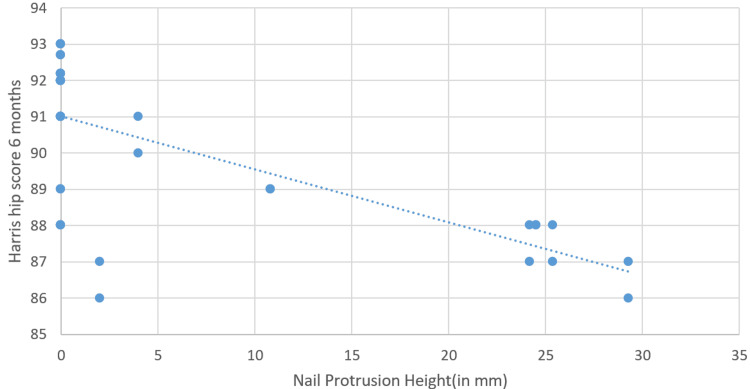
Correlation between nail protrusion height and Harris Hip Score (6 months).

At six months, Pearson’s correlation coefficient (𝑟) was −0.210, indicating a weak negative correlation (Figure 7). This suggests a slight tendency for the HHS to decrease as NPH increases, but the relationship is not strong. The p-value of 0.193 suggests that this correlation is not statistically significant.

## Discussion

Pertrochanteric femur fractures account for around half of all hip fractures [3]. Fractures of the femur can vary as per the nature of injury, how the bone is fractured, and the location of the fracture [7]. According to Cummings et al., the higher incidence of stable fractures in women, especially older women, can be attributed to the increased prevalence of osteoporosis and the higher likelihood of sustaining low-energy traumas, such as falls from a small height [8].

The NPH in patients in our study ranges widely from no protrusion (0.00 mm) to 29.30 mm, with a mean of 6.01 mm with an SD of 10.39 mm. The broad range of protrusion heights suggests that there is no standard protrusion height. This variability could be attributed to anatomical variations and the specific characteristics of the fractures. Excessive nail protrusion has been associated with complications such as trochanteric bursitis, irritation of the soft tissues, and increased pain, potentially affecting the patient's functional outcome and comfort according to Barton et al. [9].

Parker’s ratio was found to be directly proportional to the nail protrusion. It averages 41.63% with an SD of 8.84% and ranges from 18.18% to 63.13%. Parker and Handoll noted that effective rehabilitation is critical for improving functional outcomes [10], and the wide range in Parker's Ratio observed in the current study aligns with their findings, highlighting the variability in patient recovery.

The improvement in HHS indicates significant improvements in patients' hip function over time following surgery. The initial mean HHS of 32.69 immediately postoperative reflects the immediate impact of surgery, where patients are likely experiencing pain, limited mobility, and the acute effects of the surgical intervention. This low score is expected as patients are in the initial stages of recovery, which is similar to the study done by Singh et al. [11].

There is a statistically significant association between Parker's ratio and NPH, suggesting a potential correlation between these variables. The observed association aligns with the study by Hagen et al., which investigated the relationship in the context of hip biomechanics [12].

There is a potentially significant association between sex and NPH, with a p-value of approximately 0.049, which suggests that in female patients, the nail protrusion is greater. Previous research by Ritter et al. documented sex-related disparities in orthopedic parameters, including bone morphology, joint biomechanics, and implant performance [13]. Studies have reported that differences in pelvic anatomy and hip morphology between male and female individuals may influence orthopedic outcomes such as implant positioning and stability, which could potentially impact NPH [14].

In our study, the Pearson correlation coefficient of -0.303 indicates a weak negative relationship between Parker's ratio and HHS (postoperative). This suggests that as Parker's ratio increases, the HHS tends to decrease as well, reflecting a slightly inferior postoperative hip function. There was no change in Parker’s ratio over the duration and follow-up of six months. The Pearson correlation coefficient of -0.468 indicates a moderate negative linear relationship between NPH and HHS (postoperative). This indicates that as NPH increases, reflecting a greater degree of protrusion of the implant, the HHS tends to decrease, indicating poorer postoperative hip function. Consistent with the current findings, a study by Hu et al. has suggested that optimal implant positioning, as indicated by parameters like Parker's ratio and nail protrusion, is associated with improved functional outcomes and patient satisfaction following PFNA2 [6]. In their study, they found that 87.8% of patients had nail protrusion out of 51 patients. In our study, out of 40 patients, 14 patients (35%) had nail protrusion. In 25% of cases (10 out of 40 patients), NPH was >10 mm. Out of these 10 patients, only four were male, and the rest were female. The average protrusion height was 6.01 mm with a SD of 10.39 mm (male average 3.746 mm with 8.95 mm SD and female average 10.214 mm with 11.83 mm SD). In 10% of cases (n=4), NPH was < 5 mm.

There was no change in the nail protrusion over the course of follow-up, which was consistent with Parker’s Ratio. There is also a steady rise in the HHS over time. After six months, all the patients had excellent HHS, which shows that there was no further collapse of the fracture. This indicates that PFNA2 is a magnificent implant for stable and unstable pertrochanteric femur fractures. For a better understanding of nail protrusion and its clinical correlation, more studies should be carried out with a larger sample size and a longer follow-up period.

Limitations

One of the primary limitations of this study is the relatively small sample size of 40 patients, which may not adequately represent the broader population and limits the generalizability of the findings. The follow-up period of six months may be insufficient to capture long-term complications or functional outcomes associated with PFNA2 fixation. Moreover, the study does not include a control group treated with alternative fixation methods, making it difficult to directly compare the efficacy of PFNA2 with other implants.

## Conclusions

Pertrochanteric femur fracture is common in day-to-day life, with a bimodal age distribution. In elderly patients, it is common due to osteoporosis. Our study concludes that PFNA2 is a magnificent implant for treating pertrochanteric femur fractures. All patients in this study had an excellent follow-up score. Some patients, who were mainly female, had significant nail protrusion over the greater trochanter, with a higher Parker’s ratio, and had mild lateral hip pain. The pain was not significant and did not affect the patient’s day-to-day activities. This could be improved with the central placement of the helical blade. We do not recommend any changes in nail design; rather, we suggest proper helical blade placement to avoid nail protrusion. These variations due to sex and age should be studied further to establish any changes in implant design. Further studies with larger sample sizes and longer follow-ups should be carried out for more definite results.
